# A new therapy (MP29-02*) effectively controls nasal symptoms of seasonal allergic rhinitis irrespective of severity

**DOI:** 10.1186/2045-7022-3-S2-O16

**Published:** 2013-07-16

**Authors:** Glenis Scadding, David Price, Jean Bousquet, Peter Hellings, Wytske Fokkens, Ullrich Munzel, Claus Bachert

**Affiliations:** 1 The Royal National Throat, Nose and Ear Hospital, London, UK; 2 University of Aberdeen, Dept of General Practice & Primary Care, Aberdeen, UK; 3 Hopital Arnaud de Villeneue University Hospital, Montpellier, France; 4 University Hospitals Leuven, Dept of Otorhinolaryngology, Head and Neck Surgery, Leuven, Belgium; 5 Academic Medical Center, Dept of Otorhinolaryngology, Amsterdam, the Netherlands; 6 Meda Pharma, Biostatistics & Market Access, Bad Homburg, Germany; 7 Ghent University Hospital, Dept of Oto-rhinolaryngology, Ghent, Belgium

## Background

It is important to show efficacy in allergic rhinitis (AR) patients regardless of symptom severity since most AR patients presenting to a doctor have moderate-to-severe disease.

## Objective

To assess the efficacy of MP29-02* (a novel intranasal formulation of azelastine hydrochloride [AZE] and fluticasone propionate [FP]) compared to AZE, FP or placebo nasal sprays in seasonal AR (SAR) patients according to severity.

## Methods

610 patients (>= 12 years old) with moderate-to-severe SAR were randomized into a double-blind, placebo-controlled, 14-day, parallel-group trial to receive MP29-02*, commercially-available AZE or FP nasal sprays or placebo (all given as 1 spray/nostril bid; total daily dose: 548 µg AZE; 200 µg FP]). The primary efficacy variable was change from baseline in rTNSS (AM + PM). This primary endpoint was assessed post-hoc according to symptom severity. All patients had moderate-to-severe disease. Patients were categorized into two severity groups according to their median baseline rTNSS. Those with a baseline rTNSS > 18.9 points were defined as more severe and those with a baseline rTNSS <= 18.9 points were defined as less severe.

## Results

MP29-02* was significantly superior to either FP or AZE in alleviating patients' rTNSS regardless of disease severity. For those patients with less severe disease (<=18.9) MP29-02* reduced the rTNSS from baseline by -4.68 compared to -3.21 for FP (Diff: -1.46; 95% CI -2.68, -0.25; p=0.0188), -2.41 for AZE (Diff: -2.26; 95% CI -3.42, -1.10; p=0.0002) and -1.16 for placebo (Diff: -3.51; 95% CI: -4.78, -2.24; p<0.0001), corresponding to a relative treatment difference of 42% vs FP and 64% vs AZE. Patients with more severe disease (>18.9) experienced a -6.24 point reduction in their rTNSS with MP29-02*, significantly more than -4.73 with FP (Diff: -1.52; 95% CI -2.99, -0.04; p=0.0436), -4.11 with AZE (Diff: -2.13; 95% CI: -3.55, -0.71; p=0.0035) and -3.18 with placebo (Diff -3.06; 95% CI -4.34, -1.77; p<0.0001). For these more severe patients, the relative treatment effect was 49% to FP and 70% to AZE.

## Conclusion

MP29-02* provided benefits for all patients, offering significantly greater relief from nasal symptoms compared to two firstline therapies regardless of disease severity and is the drug of choice for the treatment of AR.

*Dymista

